# Bio-Based Furan-Polyesters/Graphene Nanocomposites Prepared by In Situ Polymerization

**DOI:** 10.3390/polym13091377

**Published:** 2021-04-23

**Authors:** Laura Sisti, Grazia Totaro, Annamaria Celli, Loris Giorgini, Simone Ligi, Micaela Vannini

**Affiliations:** 1 Dipartimento di Ingegneria Civile, Chimica, Ambientale e dei Materiali, DICAM, Università di Bologna, via Terracini 28, 40131 Bologna, Italy; laura.sisti@unibo.it (L.S.); annamaria.celli@unibo.it (A.C.); micaela.vannini@unibo.it (M.V.); 2 Dipartimento di Chimica Industriale ‘Toso Montanari’, Università di Bologna, Viale Risorgimento 4, 40136 Bologna, Italy; loris.giorgini@unibo.it; 3 Graphene-XT srl Via d’Azeglio, 40123 Bologna, Italy; info@graphene-xt.com

**Keywords:** graphene nanocomposites, furan-based polyesters, dynamic mechanical thermal properties

## Abstract

In situ intercalative polymerization has been investigated as a strategic way to obtain poly(propylene 2,5-furandicarboxylate) (PPF) and poly(hexamethylene 2,5-furandicarboxylate) (PHF) nanocomposites with different graphene types and amounts. Graphene (G) has been dispersed in surfactant stabilized water suspensions. The loading range in composites was 0.25–0.75 wt %. For the highest composition, a different type of graphene (XT500) dispersed in 1,3 propanediol, containing a 6% of oxidized graphene and without surfactant has been also tested. The results showed that the amorphous PPF is able to crystallize during heating scan in DSC and graphene seems to affect such capability: G hinders the polymer chains in reaching an ordered state, showing even more depressed cold crystallization and melting. On the contrary, such hindering effect is absent with XT500, which rather induces the opposite. Concerning the thermal stability, no improvement has been induced by graphene, even if the onset degradation temperatures remain high for all the materials. A moderate enhancement in mechanical properties is observed in PPF composite with XT500, and especially in PHF composite, where a significative increase of 10–20% in storage modulus E’ is maintained in almost all the temperature range. Such an increase is also reflected in a slightly higher heat distortion temperature. These preliminary results can be useful in order to further address the field of application of furan-based polyesters; in particular, they could be promising as packaging materials.

## 1. Introduction

Nowadays, there is a growing interest in the preparation of new chemicals and materials based on renewable resources, as biomass-derived fuel and chemicals are a promising alternative to fossil-based materials. Chemicals from vegetable feedstocks like sugars, vegetable oils, organic acids, glycerol, and others have been proposed as monomers for polymers production [1,2,3,4]. 2,5-furan dicarboxylic acid (FDCA) in particular, has been selected as one of the most important building blocks or top value-added chemicals derived from biomass by the U.S. Department of Energy [5]. The main structural feature related with FDCA is the close similarity to its aromatic counterpart, the terephthalic acid (TA), one of the most industrial employed fossil-based monomer (for example, for poly(ethylene terephthalate) production). Hence polymers derived from FDCA are expected to substitute the petrochemicals based on TA: poly(ethylene 2,5-furandicarboxylate) is predicted to be commercially available in 2023 [6]. Currently, most reported synthetic routes to FDCA are based on the oxidation of 5-hydroxymethyl-2-furaldehyde, which can be obtained by cyclodehydration of C6 sugars like fructose and glucose [7]. A synthesis from 5-hydroxymethylfurfural is also reported [8]. The promising developments on the FDCA synthesis have led to the lab scale synthesis of several FDCA-based polyesters including poly(ethylene 2,5-furandicarboxylate) (PEF), poly(propylene 2,5-furandicarboxylate) (PPF), poly(butylene 2,5-furandicarboxylate) (PBF), and others along with some FDCA-based copolyesters [9,10]. The furanic-aliphatic family seems to be one of the most investigated in recent years [11,12,13] and the progress on the synthesis and characterization of FDCA-based polyesters has been recently reviewed by Zhang et al. [14].

Concerning composites, the state of the art reports furan-based resin composites with clays, basalt, glass, or flax fibers [15,16,17,18]. Recent papers reported about PPF or PBF composites with montmorillonite clays [19,20], or graphene [21,22], PEF with nanocellulose or nanosized silica and titanium dioxide [23,24], PHF with graphene platelets and/or silica nanoparticles [25]. Therefore, studies in this field may contribute to further develop the furanic-aliphatic polyesters family.

Among fillers commonly used in composites, graphene, with its combination of extraordinary physical properties and ability to be dispersed in various polymer matrices, is a promising candidate for a new class of furan-based polymer nanocomposites. Graphene is an atomically thick, two-dimensional sheet composed of sp^2^ carbons organized in a honeycomb structure. It has been considered the building block of all other graphitic carbon allotropes such as graphite, fullerenes, carbon nanotubes (CNT), and nanoribbons, even if, however, these carbon allotropes are not synthesized from graphene (except for nanoribbons). Graphite indeed is a naturally occurring material, single-walled CNT was first synthesized in 1991 following the discovery of fullerenes in 1985. With Young’s modulus of 1 TPa and ultimate strength of 130 GPa, single-layer graphene is the strongest known material. Moreover, single-layer graphene has very high electrical conductivity, up to 6000 S/cm, and a thermal conductivity of 5000 W/mK. These excellent properties, in addition to extremely high surface area (2600 m^2^/g) and gas impermeability indicate graphene as the perfect candidate for improving mechanical, electrical, thermal, and gas barrier properties of polymers [26]. Graphene indeed has been used to fabricate high performing materials with novel functionalities for electric conductive composites, ultrasensitive sensors, super-capacitor electrodes, thermally stable, and mechanically reinforced materials [26,27,28,29,30]. Successful polymerizations of poly(methyl methacrylate), epoxy and poly(arylene disulfide) with graphene oxide or silicone foams and polyurethane with thermal reduced graphene oxide have been reported [26], as well as graphene nanoplatelet composites with epoxy thermosetting resin [31], nylon 6,6 [32], poly(ethylene) [33], poly(propylene) [34], silicone [35], or poly(1,4-cis-isoprene) rubber [36]. Some recent examples reported graphene and bio-based polymers as poly(butylene succinate) [37], poly(hydroxyalkanoates) [38], poly(vinyl alcohol) [26], alginate foam and hydrogels for environmental remediation [39,40].

In a previous work of the same Authors [41], it was demonstrated that stabilized water graphene suspension could be easily employed for the preparation of green composites by the in situ polymerization method. In particular, bio-based polyamide 11-graphene nanocomposites with different filler concentrations (range 0.25–3.00 wt %) were prepared and the findings showed that the nanocomposite with the highest thermal and mechanical improvement had a filler content of 0.75 wt %. Higher compositions lowered the performances, probably because of aggregation graphene nanoplatelets phenomena, causing a poor interfacial interaction with the matrix. Therefore, based on such results, a second work was developed, concerning bio-based furan polyester-graphene nanocomposites, prepared by the same procedure, in order to provide additional information on the strategy of composite preparation, on the functional properties induced by graphene suspensions and to further address the field of application of furan-based polyesters.

**Figure 1:**
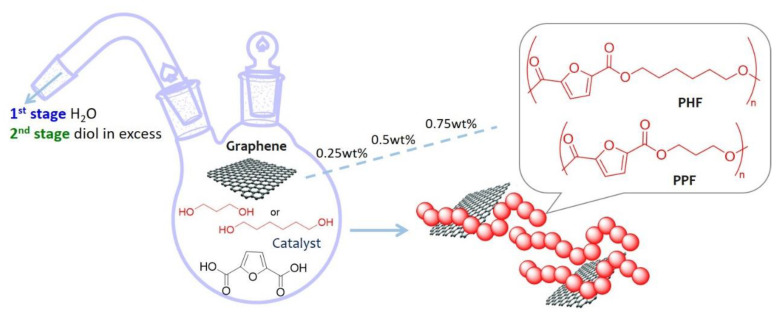
General scheme of synthesis of PPF or PHF graphene composites.

In particular, in the present work, poly(propylene 2,5-furandicarboxylate) (PPF) and poly(hexamethylene 2,5-furandicarboxylate) (PHF) have been taken into consideration. PPF is a promising sustainable alternative of poly(ethylene terephthalate) (PET), while PHF, in comparison with PEF, PPF, and PBF, shows minor tensile modulus and strength. Jiang et al. [42] indeed, reported that tensile modulus decreased with the increasing of the methylene number of aliphatic diols for furan-polyesters, therefore PHF could be a good candidate as a polymeric matrix for the preparation of composites with the aim to improve mechanical performances. Hence, PPF and PHF graphene-composites have been prepared by in situ intercalative polymerization of monomers. Such a simple procedure avoids the use of powders, employed in the melt compounding techniques, and the use of a large amount of solvent, employed for solvent casting methods, representing an advantage in term of safety and sustainability, beside the fact that a better dispersion could be achieved. Graphene (G) was dispersed in surfactant stabilized water suspensions. A second type of graphene named XT500 was tested with PPF at the highest composition (0.75 wt %), in order to evaluate the effect of the presence of oxygen on the final properties. In fact, respect to G, XT500 contains 6% of oxidized graphene and a higher tendency in forming aggregates but it does not require a surfactant to be exfoliated. Therefore, being not stable in water, it was dispersed in 1,3 propanediol. The composition range, calculated with respect to the theoretical amount in the final polymer, was 0.25–0.75 wt %. The general scheme of synthesis is reported in Figure 1. The materials have been studied in terms of molecular, morphological, thermal, and mechanical behavior by ^1^ H NMR, gel permeation chromatography (GPC), diffractometry (XRD), thermogravimetry (TGA), differential scanning calorimetry (DSC), dynamic mechanical analysis (DMTA), and scanning electron microscopy (SEM).

## 2. Materials and Methods

### 2.1. Materials

1,3-propanediol (PD, purity 98%) and 1,6-hexanediol (HD, purity 97%) were purchased from Sigma-Aldrich; 2,5-furandicarboxylic acid (FDCA, purity 97%) was provided by Aldrich; antimony trioxide (Sb_2_O_3_, purity 99%) was supplied by Carlo Erba. The reactants have been used as received. Graphene (G) dispersions in water, exfoliated using a proprietary surfactant, were kindly provided by Graphene-XT (Bologna, Italy). The industrial grade graphene G from Graphene-XT had an average lateral width of about 0.2 μm and 2–10 nm thickness. The concentration of the undisclosed suspending agent was below 1% w/w; for further information, refer to the technical data sheet [43]. Graphene XT500 (average flakes size below 2 μm, 2–8 nm thickness, with a 6% of oxidized graphene), dispersed in PD, was provided by Graphene-XT (Bologna, Italy).

### 2.2. Melt Polycondensation of poly(propylene 2,5-furandicarboxylate) (PPF) and In Situ Polymerization of PPF Graphene Composites

The polycondensation procedure was described in previous papers [11,44,45]. 28.1 g (0.180 mol) of FDCA, 24.6 g (0.324 mol) of PD and 320 mg (1.1 10^−3^ mol) of Sb_2_O_3_ corresponding to 1 wt % respect to the theoretical final polymer, were charged into a glass reactor (250 mL capacity). The ratio PD/FDCA was 1.8. The reactor was closed with a three-necked flat flange lid equipped with a heating band (T = 80 °C), a mechanical stirrer and a torque-meter (which gave an indication of the viscosity of the reaction melt). The system, under nitrogen flux, was then connected to a water-cooled condenser and immersed in a thermostatic salt-bath at 190 °C, while the stirrer was switched on at 200 rpm. The temperatures of band and bath were gradually increased up to 100 and 220 °C, respectively. All the water distilled off after less than 2 h. The reactor was then connected to a liquid nitrogen-cooled condenser. Dynamic vacuum was applied in 2 h down to 0.14 mbar, while the temperature was gradually increased up to 250 °C for the salt-bath and 120 °C for the band. When the torque of the melt was around 4 mN, a viscous, brown and transparent melt was discharged from the reactor. The molecular structure of PPF was confirmed by ^1^H-NMR.

**Figure 2:**
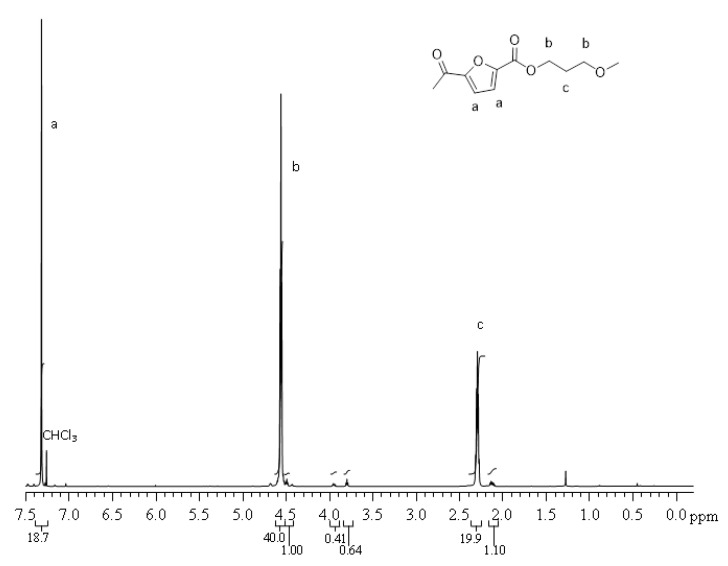
^1^ H NMR spectrum of PPF.

^1^ H NMR (CDCl_3_/d-TFA, 80/20 (*v/v*), 600 MHz): δ 2.28 (quint, 2H, C^c^H_2_, J = 6.15 Hz), 4.55 (t, 4H, C^b^H_2_, J = 6.15 Hz), 7.31 (s, 2H, C^a^H). The carbon labels are referred to the chemical structure reported in Figure 2.

The composites have been similarly prepared by adding the dispersion containing graphene into the reactor. The compositions have been calculated in wt % respect to the theoretical final polymer. The samples are denoted PPF-XYw(d), where X is the wt % of graphene, Y indicate the graphene type G or XT500, and w or d indicates a water or diol dispersion.

### 2.3. Melt Polycondensation of poly(hexamethylene 2,5-furandicarboxylate) (PHF) and In Situ Polymerization of PHF Graphene Composite

28.3 g (0.181 mol) of FDCA, 25.7 g (0.218 mol) of HD and 407 mg (1.4 10^−3^ mol) of Sb_2_O_3_, were charged into a glass reactor. The ratio HD/FDCA was 1.2. The reactor was closed with a three-necked flat flange lid equipped with a heating band (T = 80 °C), a mechanical stirrer, a torque-meter (which gave an indication of the viscosity of the reaction melt). The system was kept under nitrogen flow, then connected to a water-cooled condenser and immersed in a thermostatic salt-bath at 180 °C, while the stirrer was switched on at 200 rpm. The temperature was gradually increased up to 220 °C, as well as the heating band (100 °C), until almost all the water distilled off, after less than 2 h. The reactor was then connected to a liquid nitrogen-cooled condenser. Dynamic vacuum was applied in 2 h down to 0.23 mbar, while the temperature was increased up to 250 °C and the lid was set at 120 °C. When the torque of the melt was around 8 mN, a viscous, dark brown and transparent melt was discharged from the reactor. The molecular structure of PHF was confirmed by ^1^H-NMR.

**Figure 3:**
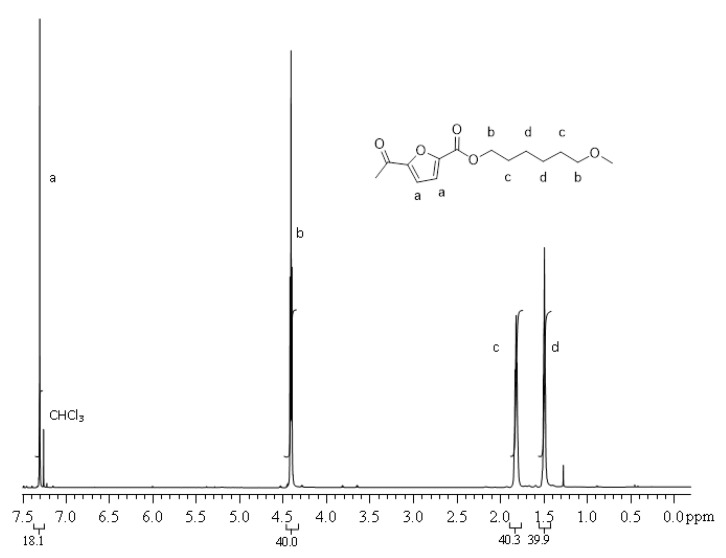
^1^ H NMR spectrum of PHF.

^1^ H NMR (CDCl_3_/d-TFA, 80/20 (V/V), 600 MHz): δ 1.49 (bs, 4H, C^d^H_2_), 1.82 (bs, 4H, C^c^H_2_), 4.41 (t, 4H, C^b^H_2_, J = 6.74 Hz), 7.30 (s, 2H, C^a^H). The carbon labels are referred to the chemical structure reported in Figure 3.

The composite was similarly prepared by adding the water dispersion containing G into the reactor. The sample is named PHF-0.75Gw and corresponds to 0.75 wt % of graphene respect to the theoretical final polymer.

### 2.4. Measurements

^1^ H NMR spectra were recorded on Varian Inova 600 spectrometer (chemical shifts are in part per million downfield from TMS); the solvent used was CDCl_3_.

Gel permeation chromatography (GPC) measurements were performed on a HP 1100 Series using a PL gel 5 µm Minimixed-C column with a solution of CHCl_3_ and 1,1,1,3,3,3-hexafluoroisopropanol (HFIP) 95/5 as eluent and to dissolve polymer samples. A UV detector was used and a calibration plot was constructed with polystyrene standards.

Thermogravimetric analysis (TGA) was performed in nitrogen atmosphere, using a Perkin Elmer TGA7 apparatus (gas flow 40 mL/min) at 10 °C min^−1^ heating rate from 50 to 900 °C. The onset degradation temperatures (T_onset_) were taken from the intersections of the tangents of the initial points and the inflection points. The 10% mass loss temperatures (T^10^_D_) and the temperature at maximum process rate (T_max_) were also measured.

The calorimetric analysis (DSC) was carried out by means of a Perkin-Elmer DSC6. Measurements were performed under nitrogen flow. In order to delete their previous thermal history, the PPF samples (ca. 10 mg) were first heated at 20 °C min^−1^ from 20 to 220 °C, kept at high temperature for 2 min and then cooled to 0 °C at 10 °C min^−1^. After this thermal treatment, the samples were analyzed by heating from 0 °C to 220 °C at 10 °C min^−1^ (second scan). The PHF samples were heated at 20 °C min^−1^ from 0 to 180 °C, kept at high temperature for 2 min, then cooled to −20 °C at 10 °C min^−1^, and heated from −20 °C to 180 °C at 10 °C min^−1^ (second scan). During the cooling scan the crystallization temperature (Tc) and the enthalpy of crystallization (∆Hc) were measured. During the second scan, the glass transition temperature (T_g_), the melting temperature (T_m_) and the enthalpy of fusion (∆H_m_) were measured. T_g_ was taken as the midpoint of the heat capacity increment associated with the glass-to-rubber transition.

The X-ray diffraction (XRD) analysis was carried out at room temperature by means of a PANalytical X’Pert PRO diffractometer equipped with an X’Celerator detector. Data were acquired by exposing the samples to Cu-Kα X-ray radiation, which has a characteristic wavelength (λ) of 1.5418 Å. X-rays were generated from a Cu anode supplied with 40 kV and a current of 40 mA. The 2θ plots of X-ray spectra have been obtained over 2θ range of 2.1–35°. For each step of 0.067°, the signal has been counted for 41 s, to increase its intensity. PPF film samples, pressed at 200 °C were analyzed. PHF samples, were pressed at 100 °C, obtaining samples with almost 1 mm of thickness and a smooth and regular surface to analyze.

Physical and mechanical properties were determined using a Rheometric Scientific DMTA IV Dynamic Mechanic Thermo analysis instrument with a dual cantilever testing geometry. Typical test samples were bars obtained by injection molding at 190 °C using a Minimix Molder with PHF. PPF sample bar have been obtained by compression molding at 200 °C. The analysis was carried out at a frequency of 3 Hz, at 3 °C min^−1^ in the temperature range starting from −120 °C to 110 °C.

Fracture surfaces of samples were obtained under cryogenic fracture by liquid nitro-gen to avoid plastic deformations. Sections were analyzed by scanning electron microscopy (Phenom ProX, samples gold coated) to investigate their morphology.

## 3. Results and Discussion

In a previous work by the same Authors [41], it was demonstrated that stabilized water graphene suspension could be easily employed for the preparation of bio-based polyamide nanocomposites by the in situ polymerization method. Therefore, in the current work, bio-based furan polyester-graphene nanocomposites have been prepared by a similar procedure, in order to provide supplementary information about this strategy of preparation of composites through a chemical approach that does not require solvents. Moreover, the use of graphene as filler can address new fields of application of furan-based polyesters. PPF and PHF composites with a graphene loading range of 0.25–0.75 wt %, have been prepared, as higher contents were proven to confer decreased performances, due to graphene nanoplatelets aggregation phenomena [41]. Graphene (G), was furnished in surfactant stabilized water suspensions while XT500 was dispersed in 1,3-propanediol (PD), without any surfactant, and used for the highest composition (0.75 wt %). More in detail, PPF composites have been prepared with 0.25, 0.50, and 0.75 wt % of G, as well as with 0.75 wt % of XT500 dispersed in PD; PHF composite has been prepared with 0.75 wt % of G in surfactant stabilized water suspension. All the prepared polymeric samples are summarized in Table 1.

The molecular structure was checked by ^1^ H NMR. PPF and PHF homopolymers spectra are showed in Figure 2 and Figure 3, respectively. The profiles are coherent with literature [42]. In Figure 2, around 3.8 ppm, it is possible to observe a small amount of ether bridges (<2 mol %), already reported for PPF [11].

The samples have high molecular weight within the range 50,000–70,000 g/mol (Table 1) with a polydispersity around ≈ 4, quite high in comparison to common polyesters (≈2–3). Vannini et al. [11] reported a polydispersity of 3 for a PPF with high Mw (55,000) prepared through a similar procedure. Knoop et al. [46] obtained lower polydispersity values (around 2) for a series of furan-based polyester with Mw into the range 15,000–30,000 but their procedure required longer reaction times and the polycondensation was followed by a solid state post condensation. On the other hand, Jiang et al. [42] obtained high Mw (90,000) with a low polydispersity (1.5) for a PPF synthesized through classical polycondensation. The GPC profiles of the samples here prepared indicate a multimodal distribution with minor peaks after and before the principal peak (profiles not showed). Such minor peaks correspond to groups of molecules with lower and higher hydrodynamic volume. This indicates the presence of different populations of polymeric chains, probably determined by the catalyst or by some side-chains reactions or by the procedure conditions. Among all samples, PPF-0.75XT500d presents the highest Mw and polydispersity, possibly because graphene is dispersed in 1,3-propanediol (without any surfactant), which is also a co-monomer in PPF polymerization. Thus, in this case the esterification could be promoted, possibly leading to a widening in the molecular weight distribution.

**Figure 4:**
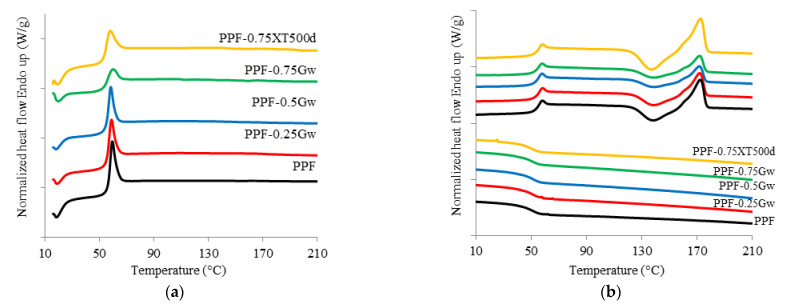
(**a**) Calorimetric curves of PPF and PPF composites with graphene, first heating scan; (**b**) second heating scan and cooling scan.

**Figure 5:**
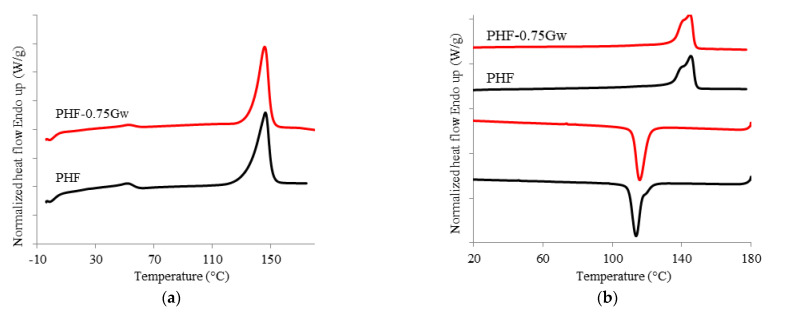
(**a**) Calorimetric curves of PHF and PHF-075Gw, first heating scan; (**b**) second heating scan and cooling scan.

The thermal behavior of the investigated samples was assessed. Calorimetric data are summarized in Table 1 while the corresponding thermograms are shown in Figure 4 and Figure 5. PPF is an amorphous polymer, with a glass transition temperature (T_g_) of 53 °C. During the cooling scan from the melt at 10 °C/min, PPF is unable to crystallize; it only presents, during the second heating scan (at 10 °C/min) a cold crystallization process at 138 °C and a melting peak at 172 °C. All the PPF composites with G are substantially amorphous too, but some observations can be done. The samples PPF-Gw during the second heat scan present low enthalpy values (ΔH_cc_ and ΔH_m_) with respect to the homopolymer. Moreover, the shape of the peaks become broader, showing cold crystallization and melting processes more and more depressed. The polymer chains in such samples have even more difficulties in reaching an ordered state; therefore, Gw hinders the crystallization of the matrix occurring during heating. This behavior can be due to the presence of surfactant used to stabilize the G suspension. On the other hand, in PPF-0.75XT500d, cold crystallization and melting processes are more intense than those of PPF and of the corresponding PPF-0.75Gw. The graphenic sample XT500 seems to favor the rearrangement of the polymer chains during heating, probably due to the absence of surfactant and/or to the presence of 6 wt % of graphene oxide, and/or to the larger lateral width respect to G. In contrast, PHF is semicrystalline (Figure 5): it crystallizes during the cooling scan at 114 °C (T_c_) and melts at 145 °C (T_m_). The glass transition temperature is 13 °C. No significant differences are present in the composite: graphene does not seem to affect the thermal behavior of PHF. The DSC data are consistent with literature [10,11,47]. Moreover, as it can be observed from both Figure 4b and Figure 5b, the melting shape is complex, then more than one crystal type is formed or melting/recrystallization processes occur.

Concerning the T_g_, no substantial changes respect to both PPF and PHF were highlighted. Graphene does not seem to affect the mobility of the chains neither as plasticizing neither as nucleating agent.

**Figure 6:**
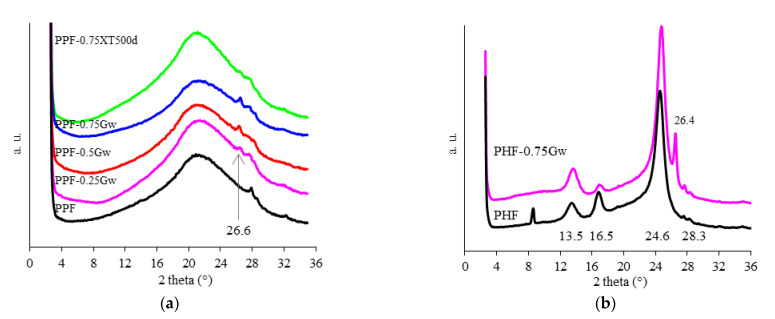
Diffractometric curves of (**a**) PPF samples (the arrow indicates the reflection attributed to (002) diffraction peak of graphene) and (**b**) PHF samples.

Figure 6a shows the XRD diffractograms of PPF samples. A large amorphous halo is visible, as well as the catalyst Sb_2_O_3_ around 2θ = 28°. The reflection attributed to (002) diffraction peak of graphene, at 26.6° [48], is visible in all composites. Figure 6b shows the diffractometric curves of PHF samples, proving the semicrystalline nature of such samples. PHF presents main reflections at 13.5°, 16.9°, and 24.6°, corresponding to (110), (010), and (111) planes, respectively [14]. The catalyst Sb_2_O_3_ is similarly visible around 2θ = 28°, as well as the (002) diffraction peak of graphene in PHF-0.75Gw [48].

**Figure 7:**
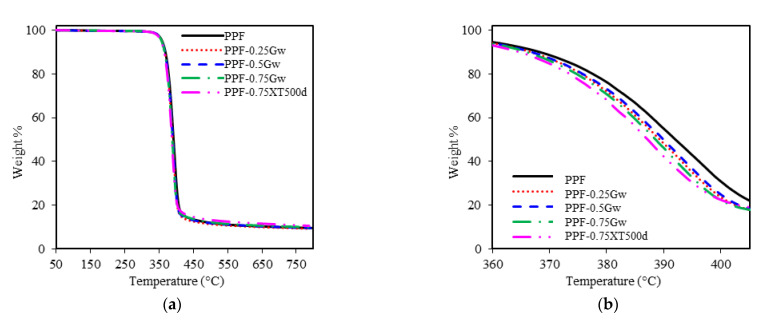
(**a**) Thermogravimetric curves of PPF samples; (**b**) zoomed region.

**Figure 8:**
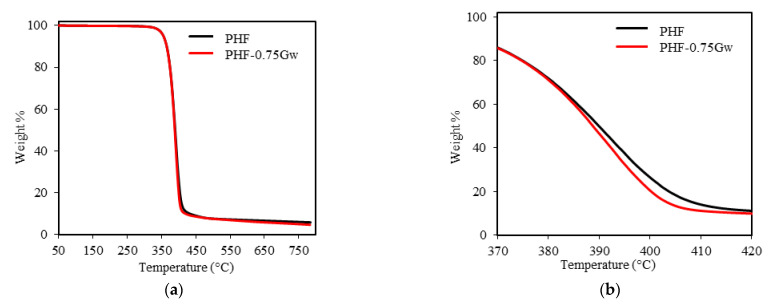
(**a**) Thermogravimetric curves of PHF samples; (**b**) zoomed region.

The thermal stability of homopolymers and their nanocomposites with graphene was investigated by TGA under inert nitrogen atmosphere. The thermogravimetric curves are shown in Figure 7 and Figure 8, while the thermogravimetric data are summarized in Table 2. As it can be seen from figures, all the samples decompose in one single step. PPF has an onset degradation temperature of 371 °C and a maximum rate of decomposition at 393 °C. PHF presents similar values of T_onset_ (369 °C) and T_max_ (391 °C). T_onset_, T_max_, and T^10^_D_ of all the composites remain almost similar to the respective homopolymers, no significant differences are present. Thus, graphene does not seem to improve the thermal stability of the polyester matrix: indeed, decomposition curves shift toward slightly lower temperature region. On the contrary, slight retardation in decomposition were reported by Paszkiewicz et al. for PPF composites with few layer graphene [21], while more consistent thermal stability improvements were reported in case of different matrices such as poly(styrene) (PS), poly(vinyl alcohol) (PVA), poly(methyl methacrylate) (PMMA) [26]. The non-volatile fractions, corresponding to residual masses for PPF and PHF composites, are ≈10 and 5 wt %, respectively. These data, not affected by the presence of graphene, are consistent with literature [7,42] and are due to the decarboxylation of FDCA [49].

**Figure 9:**
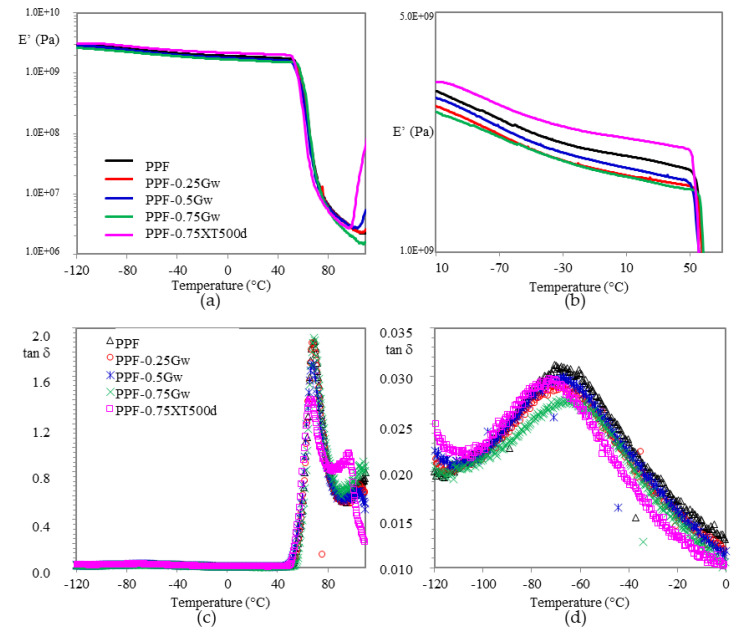
(**a**) Storage modulus E’ vs. T in PPF samples and (**b**) zoomed region; (**c**) tan δ vs. T and (**d**) zoomed region.

**Figure 10:**
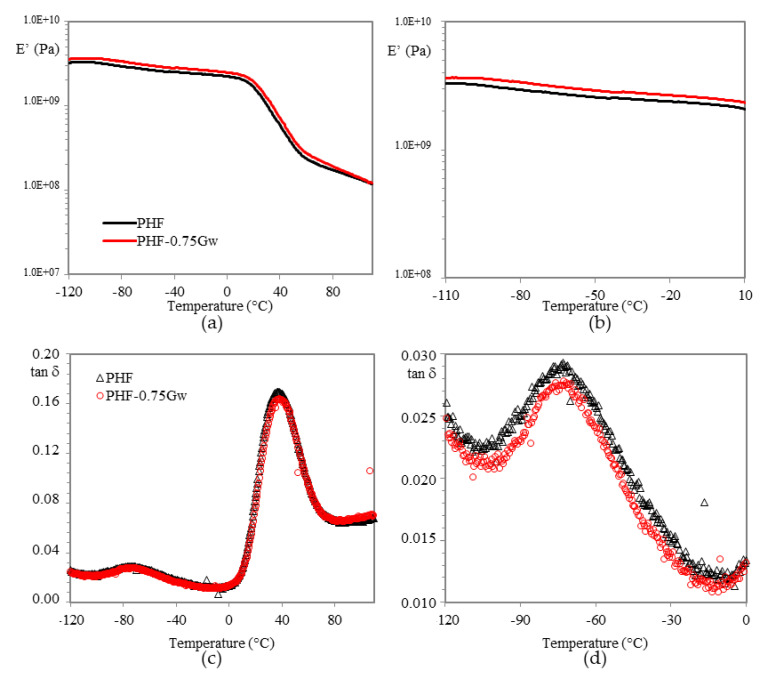
(**a**) Storage modulus E’ vs. T in PHF composites and (**b**) zoomed region; (**c**) tan δ vs. T and (**d**) zoomed region.

Temperature and frequency dependent mechanical relaxation data for amorphous PPF and semicrystalline PHF were recorded via storage modulus (E′) and tan δ, which is the ratio of the loss modulus to storage modulus. Such data can provide indications about the mechanical reinforcement, which is correlated to a good interface interaction filler/matrix due to the presence of van der Waals forces or π–π interactions [50]. In Figure 9 and Figure 10 the changes of E’ and tan δ as function of temperature for all the investigated samples are plotted. Concerning the PPF samples, E′ values of graphenic composites decrease respect to the homopolymer (Figure 9a,b). This indicates that no mechanical reinforcement of the matrix has been induced by graphene, probably because of the presence of surfactant. In fact, only PPF-0.75XT500d presents, from low temperature up to room temperature, a moderate enhancement in storage modulus E’, quantifiable as 6–12%. The larger storage modulus for such composite indicates a slight mechanical reinforcement of the matrix due to a better interfacial interaction filler/polymer, possibly ascribable to the absence of surfactant or to the chemical environment, which could help in preventing the restacking of the sheets, being PD a co-monomer during PPF polycondensation.

Similarly, a slight reinforcing effect of graphene in E’ was reported with PMMA [51]. The authors reported also an increase in tan δ peak due to a restricted mobility of the PMMA chains through strong chemical interactions between the polar groups of the polymer and the remaining oxygen functionality of the graphene [51]. Akhina et al. obtained an increase in storage modulus below the glass transition temperature and a slight shift in tan δ peak, in poly(vinyl chloride) (PVC)/reduced graphene oxide (RGO) nanocomposites, due to van der Waals force of attraction between RGO and PVC [52]. A similar behavior is reported by Le et al. [53] in some epoxy/polyester blends with graphene nanoplatelets. Paszkiewicz et al. [21] reported the tensile properties of PPF nanocomposites with few layer graphene, and a slight increase in Young’s modulus (about 5%) and 200% increase in elongation at break was achieved with 0.3 wt % of graphene.

In the zoomed image of Figure 9d, at around −70 °C, it is also visible a β-relaxation. The low intensity of β-relaxation, respect for example to that of PET [54] is attributed to the high rigidity of the furan ring, which reduces the segmental motions and is strictly connected to a reduced diffusion inside the material, resulting in improved barrier properties of PPF [11]. By observing all the curves, the slight decrease in PPF-0.75Gw could indicate more pronounced barrier properties.

For all samples, the glass transition temperature is recognized by the large drop in storage modulus and by the corresponding peak maximum in tan δ (Figure 9c and Figure 10c). The values extrapolated and summarized in Table 2, do not highlight any substantial changes respect to the respective homopolymers. Graphene does not seem to affect the mobility of the chains neither as plasticizing neither as nucleating agent.

Above T_g_, all samples are substantially similar to the homopolymer.

Crystallization is observed by the increase in storage modulus around 100 °C for all samples. As already expressed, PPF is able to crystallize under specific conditions, as for example during DMTA scan at 3 °C/min, after the glass-rubber transition. Such phenomenon is more pronounced for PPF-0.75XT500d, probably due to a considerably higher crystallization rate induced under heating by graphene XT500, as already evidenced by DSC analysis.

In case of PHF samples (Figure 10), the composite presents an adequate increase in E’ of 10–20% respect to the homopolymer in almost all temperature range. Probably in this case a better interfacial graphene/matrix interaction was reached, indicating a mechanical reinforcement. No differences in β-relaxation are noticeable (Figure 10d).

Moreover, heat distortion temperature (HDT), corresponding to log E’ = 8.9 (1.82 MPa) according to Scobbo [55] was calculated (Table 2), in order to investigate the window of application of the materials prepared. HDT in fact, is a useful indicator to individuate the temperature at which the material starts to soften when exposed to a fixed load at elevated temperatures, thus resulting in a macroscopic measure of the stiffness of a material. PPF has an HDT of 60 °C and its composites remain almost at this value. On the other hand, PHF-0.75Gw, has a slightly higher HDT (38 vs. 34 °C) respect to the homopolymer.

**Figure 11:**
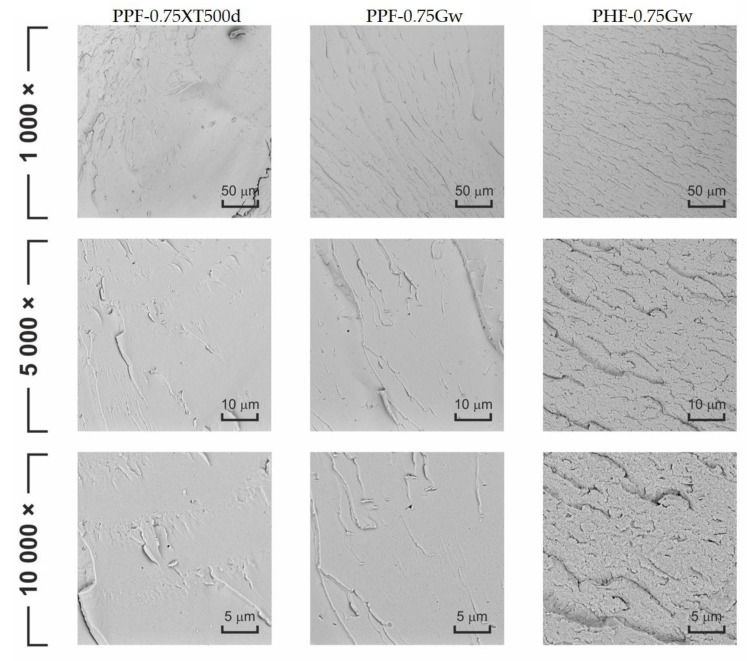
SEM images of the prepared composite products at several magnification.

Figure 11 displays the fracture surfaces of some samples after cryogenic fracture. PPF-0.75XT500d and PPF-0.75Gw show similar morphologies, characterized by brittle fracture behavior, as evidenced by flat and smooth planes. PHF-0.75Gw morphology is quite different: the surface is irregular and rough, suggesting that some plastic deformation occurs. The G carbon sheets aggregates are not identified on the cross-section surface that appears homogeneous.

## 4. Conclusions

PPF and PHF composites containing graphene were prepared by in situ polymerization. Graphene (G) was dispersed in surfactant stabilized water suspensions. For one PPF composition, a different type of graphene was also tested, XT500, dispersed in 1,3-propanediol without any surfactant. In this case, it is notably to observe that 1,3-propanediol is also a monomer for the PPF synthesis. PPF samples result to be amorphous, while PHF samples are semicrystalline materials. Graphene seems to affect the ability of PPF to crystallize during heating: the nanocomposites containing G show a depressed cold crystallization and melting processes. On the contrary, XT500 seems to help the rearrangement of the polymer chains in an ordered state.

Concerning the thermal stability, no improvement has been induced by graphene; however, the onset degradation temperatures still remain high for all the materials, around 370 °C. A moderate enhancement in mechanical properties is observed in PPF-0.75XT500d (around 6–12% of E’ increase), and especially in PHF composite, where an adequate increase of 10–20% in E’ is maintained in almost all the temperature range. Such increase is also reflected in a slightly higher HDT (38 vs. 34 °C). In terms of mechanical performances, for PPF composites 1,3-propanediol results to be a better medium to suspend graphene respect to surfactant stabilized water dispersions. PD in fact, is a monomer in PPF polycondensation and this helps in preventing the restacking of the sheets. In summary, graphene:

- affects the ability of PPF to crystallize during heating, while it does not affect the thermal transitions of PHF;
- does not affect the thermal stability neither of PPF neither of PHF;
- determines a mechanical reinforcement especially in PHF.

Such preliminary results can be useful in order to further address the field of application of furan-based polyesters. Indeed, the above materials could be potentially used for packaging application, where high barrier properties—potentially further improved by graphene—are required. Moreover, it is noteworthy that the in situ polymerization procedure avoids the use of powders, employed in the melt compounding techniques, and the use of a large amount of solvent, employed for solvent casting methods, representing an advantage in term of safety and sustainability.

## Figures and Tables

**Table 1:** Molecular characteristics and calorimetric data of PPF, PHF and their composites.

	M_w_·10^−3^ ^(1)^	M_w_/M_n_ ^(1)^	T_g_^(2)^ (°C)	ΔC_p_^(2)^ (J/g°C)	T_c_^(3)^ (°C)	ΔH_c_^(3)^ (J/g)	T_cc_^(2)^ (°C)	ΔH_cc_^(2)^ (J/g)	T_m_^(2)^ (°C)	ΔH_m_^(2)^ (J/g)
PPF	54	4.6	53	0.39	/	/	138	12	172	11
PPF-0.25Gw	64	4.6	54	0.35	/	/	138	9.1	172	9.2
PPF-0.5Gw	53	4.2	53	0.38	/	/	139	5.6	172	6.2
PPF-0.75Gw	49	4.1	53	0.31	/	/	138	6.3	172	6.4
PPF-0.75XT500d	70	5.3	53	0.41	/	/	137	16	173	15
PHF	55	3.5	13	0.05	114	38	/	/	145	35
PHF-0.75Gw	47	3.0	12	0.07	116	39	/	/	145	34

^(1)^ calculated by GPC with a 95/5 v/v CHCl_3_/HFIP solution as eluent; ^(2)^ calculated from the second heating scan at 10 °C min^−1^; ^(3)^ calculated from the cooling scan at 10 °C min^−1^.

**Table 2:** Thermal relevant data of PPF and PHF homopolymers and nanocomposites

	T_onset_^(1)^ (°C)	T_max_^(1)^ (°C)	T^10^_D_^(1)^ (°C)	Residual Mass ^(1)^ (%)	HDT ^(2)^ (°C)	T_g_^(3)^ (°C)
PPF	371	393	368	9.5	60	69
PPF-0.25Gw	371	391	367	9.2	59	68
PPF-0.5Gw	371	391	367	9.6	57	68
PPF-0.75Gw	372	390	366	9.9	59	68
PPF-0.75XT500d	370	387	365	10.6	56	67
PHF	369	391	365	5.8	34	41
PHF-0.75Gw	371	391	365	4.7	38	41

^(1)^ calculated by TGA; ^(2)^ Heat deflection temperature (HDT) calculated by DMTA, corresponding to log E’ = 8.9 (1.82 MPa); ^(3)^ T_g_ calculated by DMTA as maximum peak of tan δ.

## Data Availability

The data presented in this study are available on request from the corresponding author.
